# Intracellular ROS Protection Efficiency and Free Radical-Scavenging Activity of Curcumin

**DOI:** 10.1371/journal.pone.0026012

**Published:** 2011-10-10

**Authors:** Abolfazl Barzegar, Ali A. Moosavi-Movahedi

**Affiliations:** 1 Research Institute for Fundamental Sciences (RIFS), University of Tabriz, Tabriz, Iran; 2 Institute of Biochemistry and Biophysics, University of Tehran, Tehran, Iran; Universidad Federal de Santa Catarina, Brazil

## Abstract

Curcumin has many pharmaceutical applications, many of which arise from its potent antioxidant properties. The present research examined the antioxidant activities of curcumin in polar solvents by a comparative study using ESR, reduction of ferric iron in aqueous medium and intracellular ROS/toxicity assays. ESR data indicated that the steric hindrance among adjacent big size groups within a galvinoxyl molecule limited the curcumin to scavenge galvinoxyl radicals effectively, while curcumin showed a powerful capacity for scavenging intracellular smaller oxidative molecules such as H_2_O_2_, HO^•^, ROO^•^. Cell viability and ROS assays demonstrated that curcumin was able to penetrate into the polar medium inside the cells and to protect them against the highly toxic and lethal effects of cumene hydroperoxide. Curcumin also showed good electron-transfer capability, with greater activity than trolox in aqueous solution. Curcumin can readily transfer electron or easily donate H-atom from two phenolic sites to scavenge free radicals. The excellent electron transfer capability of curcumin is because of its unique structure and different functional groups, including a β-diketone and several π electrons that have the capacity to conjugate between two phenyl rings. Therfore, since curcumin is inherently a lipophilic compound, because of its superb intracellular ROS scavenging activity, it can be used as an effective antioxidant for ROS protection within the polar cytoplasm.

## Introduction

Under stress, human bodies produce substantial amounts of reactive oxygen species (ROS) in different tissues. In many situations, ROS (e.g., hydroxyl radicals and hydrogen peroxide) production rate will increase to level that can overwhelm non-enzymatic (e.g., glutathione, α-tocopherol, ascorbic acid, carotenoids) and enzymatic (e.g., catalase, superoxide dismutase and glutathione peroxidase) antioxidants within the cell [1], [2]. This imbalance between ROS and antioxidant levels leads to cell damage and health problems [3]. For example, chronic accumulation of ROS in the brain poses the onset and progression of Alzheimer's disease [4], [5]. One solution to this problem is to supplement the diet with antioxidant compounds present in natural sources [6]. Antioxidant supplements can reduce ROS in cells and are consequently useful for treatment of many human diseases including different types of atherosclerosis, inflammatory injuries [7], cardiovascular diseases, cancer [8], neurodegenerative diseases [9] and aging [10]. Natural antioxidants can therefore serve as a type of preventive medicine.

Curcumin, 1,7-bis(4-hydroxy-3-methoxyphenyl)-1,6-heptadiene-3,5-dione, or diferuloyl methane, the yellow pigment of turmeric and curry (*Curcuma longa Linn*), is one of the major natural spices used in Indian cuisine. This natural compound has also been used for centuries in a variety of pharmaceutical applications including use as an anti-inflammatory agent [11], [12] and as an orally available treatment for diabetes [13]. Many health benefits, such as a potential cancer chemotherapeutic properties, therapeutic benefits in several animal oxidative stress models such as for Alzheimer's disease [14], ethanol induced oxidative injuries in brain, liver, heart, kidney [15] and prevention of myocardial ischemic damage [16], have been claimed for curcumin and these have generally been attributed to its radical-trapping antioxidant properties. Experiments with curcumin have shown a little oxidative damage and very small amounts of amyloid plaques both *in vitro* [17] and *in vivo* [18], [19].

In addition to its use in traditional medicines, curcumin continues to show a lot of promise as a new remedy based on experimental efforts to identify the natural properties of this antioxidant. Curcumin has been shown to have potent antioxidant activity that effectively scavenges ROS and inhibits lipid peroxidation [20]. At present, the ability of curcumin to reduce and scavenge ROS in a highly polar solution represented by the cytoplasm is unclear, since the curcumin molecule is a highly lipophilic compound. In this study, L-6 myoblasts were used as an in vitro cell model to investigate the intracellular efficiency of curcumin as a protective agent against ROS damage. Extracellular free radical scavenging was assessed by reducing power and ESR measurements.

## Materials and Methods

Potassium ferricyanide and ferric chloride acquired from Merck. Curcumin, galvinoxyl radical, trichloroacetic acid (TCA), and cumene hydroperoxide (CHP) were purchased from Sigma Chemical Co. The 2′,7′*-*dichlorodihydrofluorescein diacetate (DCFH_2_-DA) was obtained from Molecular Probes (Eugene, OR). Fetal bovine serum was from GIBCO (Grand Island, NY). Dulbecco's modified Eagle's medium (DMEM), antibiotics and sterile plastic ware for cell culture were from Flow Laboratory (Irvine, UK).

### Cell cultures

Skeletal rat muscle cells (L-6 myoblasts) were obtained from the American Type Culture Collection (Rockville, MD). Cells were seeded in 75-cm^2^ flasks for tissue culture and grown in DMEM supplemented with 10% fetal bovine serum, 100 mg mL^−1^ streptomycin and 100 U mL^−1^ penicillin, in an atmosphere of 5% CO_2_ at 37°C. The cells were 95% confluent after5 days (about 6×10^6^ cells) and were kept in culture as myoblasts by continuous passages at pre-confluent stages.

### DCF method for detection of intracellular ROS

Measurements of intracellular ROS levels in L-6 cells were made using 2′,7′-dichlorodihydrofluoroscein diacetate (DCFH_2_-DA). DCFH_2_-DA is able to diffuse through the cell membrane and become enzymatically hydrolysed by intracellular esterases to produce non-fluorescent DCFH_2_. The oxidation of DCFH_2_ by intracellular ROS mainly H_2_O_2_, HO^•^, ROO^•^, NO^•^ and ONOO^−^ results in fluorescent DCF which stains the cells [21]. Hence, the intracellular ROS generation of cells can be investigated using the DCFH_2_-DA as an indicator to detect and quantify intracellular produced reactive oxygen species [22]. Cell samples were incubated in the presence of 10 µM DCFH_2_-DA in phosphate buffered saline (PBS) at 37°C for 30 min then washed two times with PBS and centrifuged at 1200 rpm to remove the extracellular DCFH_2_-DA. The trapped fluorescent dye (DCF) inside the cells used to evaluate and detect intracellular ROS. The fluorescence values at different conditions were monitored by excitation at 498 nm and emission 530 nm.

### MTT method for antitoxicity assay

Yellow MTT [3-(4,5-Dimethylthiazol-2-yl)-2,5-diphenyltetrazolium bromide, a tetrazole], is reduced to purple formazan in the mitochondria of living cells. The absorbance of this colored solution can be quantified by measuring at a certain wavelength (500–600 nm) by a spectrophotometer [23]. Hence, the application allows assessing the antitoxicity of curcumin based on viability of cells. When the amount of purple formazan produced by cells treated with an agent is compared with the amount of formazan produced by untreated control cells, the effectiveness of the agent in causing death of cells can be deduced. L-6 cells were seeded in 6-well plates until they reached 95–100% confluence, then they were incubated for 30 min with different concentrations of curcumin (0.0, 1.0, or 5.0 µM). Thereafter, cells induced with 5 µl (1∶100) cumene hydroperoxide (CHP) as a powerful cytotoxic ROS-generating compound [24] in a final volume of 1 ml PBS for 30 min. Control and treated cells were incubated with MTT at a final concentration of 1 mg mL^−1^ for 3 h at 37°C. The cells were then scraped off and centrifuged at 1200 rpm for 5 min. The pellet was re-suspended in 300 µL phosphate-buffered saline (PBS) and sonicated on ice for 20 seconds with an Ultrasonic W-225R, at setting 4, and centrifuged in a microfuge at 13000 rpm for 10 min. The supernatant was discarded and the water-insoluble formazan assay product was dissolved in dimethylsulfoxide (DMSO) and measured at 560 nm. A decrease in cell number results in a decrease in the amount of MTT formazan formed and a decrease in absorbance. Therfore, the percentages of cell viability were normalized and calculated with the following equation:
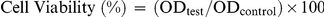



### Galvinoxyl free radical scavenging assay

This assay is based on the measurement of the scavenging ability of antioxidants toward the stable galvinoxyl radical (Gox^•^). The free radical Gox^•^ is reduced to GoxH when it reacts with hydrogen donors. This ability is evaluated by more frequently used technique (ESR). Stock solutions of galvinoxyl (10 mM in 95% EtOH) were freshly prepared before the experiments. The radical scavenging activity of different concentrations of curcumin was followed for 30 min by ESR. The solutions were drawn into glass capillaries, sealed and measured using an ESP300 instrument (Bruker Spectrospin, Karlsruhe, Germany) equipped with a high sensitivity TM110 X-band cavity. Radical spectra were recorded at room temperature, using 0.6G modulation, 1mW microwave power.

### Reducing power assay

Reducing power assessment is based on the ability of antioxidants to reduce ferric ion (Fe^3+^) to ferrous ion (Fe^2+^). This method applied to determine and analyze the electron donating potency of curcumin according to Oyaizu's method [25]. Different concentrations of curcumin were added to a mixture of potassium ferricyanide (1%) and PBS (1∶1v/v) in a final volume of 500 µl. The samples were incubated for 30 min at 50°C, then 100 µl of ferric chloride (0.1%) and 200 µl of TCA (10%) were added and mixed. A 500 µl of this solution was added to 2ml of PBS in a cuvette and the absorbance was recorded at 700nm. Samples with greater reducing power showed increased absorbance.

## Results and Discussion

### Ability of curcumin to scavenge intracellular ROS

L-6 myoblasts are good candidates and easy to use as an in vitro model for testing the ability of different types of antioxidants to scavenge intracellular ROS [26], [27]. In this study, L-6 myoblasts were used to investigate the ability of curcumin to scavenge intracellular ROS based on the DCF method. For this aim, L-6 cells pretreated with DCFH_2_-DA were exposed to the highly oxidizing agent cumene hydroperoxide (CHP), a well-known intracellular ROS generator [24].

**Figure 1:**
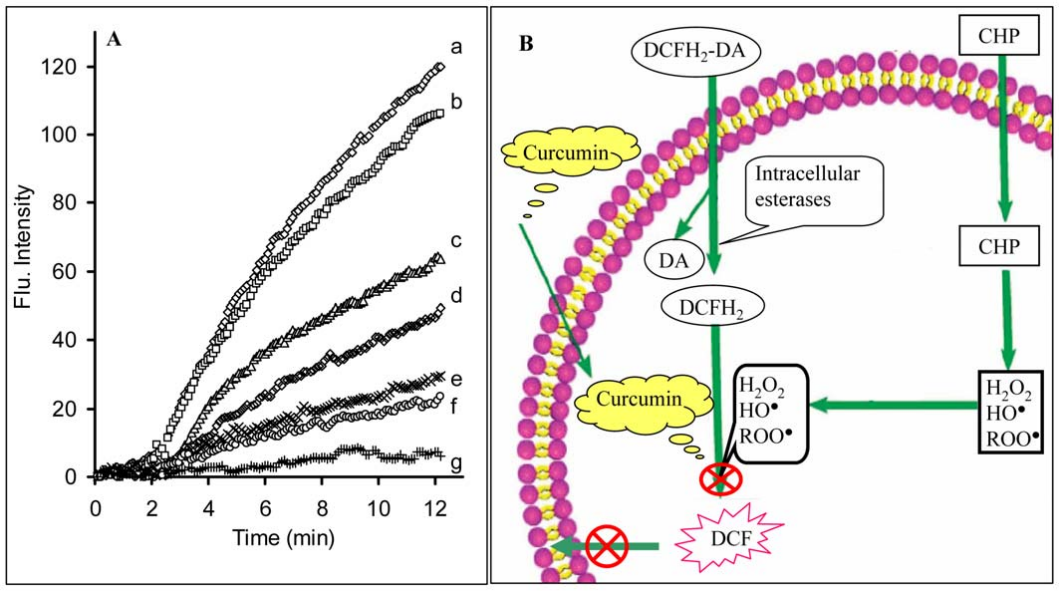
In situ analysis of intracellular ROS. A) All samples were first incubated (30 min) with different concentration of curcumin (a, 0.0; b, 0.1; c, 0.25; d, 0.5; e, 1.0; f, 2.0; g, 4.0 µM), then the DCF fluorescence intensity changes were monitored by the addition of the ROS stimulating agent cumene hydroperoxide (CHP). In the absence of CHP, no change in DCF fluorescence intensity was seen with time; however, it started increasing in presence of CHP. B) The principle of the intracellular ROS protection activity of curcumin. Curcumin diffuses easily into the cells prevents ROS production, thereby preventing oxidation of DCFH_2_ and the formation of the fluorescent DCF product.

Figure 1A indicates the intracellular ROS scavenging activity of curcumin at seven different concentrations (0.0–4.0 µM). In the absence of curcumin, CHP causes substantial oxidation of DCFH_2_ to DCF, leading an increased rate of fluorescence intensity change. Addition of curcumin gradually decreases the intracellular fluorescence intensity and completely suppresses it at a concentration of 4.0 µM. Therefore, curcumin is able to diffuse through the cell membrane into the cells, where it prevents production of different ROS compounds required to oxidize intracellular DCFH_2_ to the fluorescent DCF.

The mechanism action of curcumin as a powerful intracellular antioxidant molecule that can easily permeate through cell membrane and intercept ROS forming radicals is shown schematically in Figure 1B. The conversion of the nonfluorescent DCFH_2_-DA to highly fluoresecent DCF, occurs in several steps. DCFH_2_-DA is transported across the cell membrane and deacetylated by esterases to form the non-fluorescent DCFH_2_. This compound is trapped inside the cells. Next, DCFH_2_ is converted to DCF through the action of intracellular oxidants; H_2_O_2_, HO^•^, ROO^•^. Curcumin combats intracellular ROS generation (H_2_O_2_, HO^•^, ROO^•^), leads the amount of DCF fluorescence to decreased, as shown in Figure 1A. Consequently, the efficient suppression of intracellular ROS production by curcumin indicates that this compound enters the cells and acts with strong radical scavenging potency in the polar intracellular environment.

### Antitoxic potency of curcumin

MTT method was used to evaluate the antitoxic properties of curcumin against cytotoxic and lethal effects of CHP. The reduction of yellow MTT to purple formazan takes place only when mitochondrial reductase enzymes are active, and therefore the amount of conversion can be directly accounted for the percentage of viable (living) cells [23]. Table 1 shows that in the absence of CHP, 100% of cells are viable that reduces MTT compound. While, CHP is a highly toxic and lethal compound for L-6-cells that causes 78% of cells to die. Presence of curcumin in samples almost suppressed the lethal effects of CHP. As a powerful stressor molecule, CHP causes the generation of different intracellular ROS; H_2_O_2_, HO^•^, ROO^•^, culminating in lethal effects. It can be concluded that, the effect of curcumin in blocking the toxic and lethal effects of CHP has a high correlation with its intracellular ROS scavenging potency that has been shown in Figure 1.

**Table 1:** Effect of curcumin on viability of L-6 myoblasts exposed to cumene hydroperoxide (CHP).

*CHP*	*Curcumin (*µ*M)*	*Viable Cells (%)*
**-**	0	100 (±3)
**+**	0	22 (±4)
**+**	1	52 (±5)
**+**	5	93 (±4)

After a 30 min treatment of cells with different concentrations of curcumin (0, 1 and 5.0 µM) the cells were induced with 5 µl (1∶100) CHP. Viability was measured by the MTT assay.

**Table 2:** Comparative physical properties of curcumin, trolox and α-tocopherol.

*Properties* *	*Curcumin*	*Trolox*	*α-tocopherol*
**H-Bond Acceptors**	6	4	2
**H-Bond Donors**	2	2	1
**Rotatable Bonds**	8	1	12
**Polar H as OH**	2	2	1
**^a^SA_tot_ (A°^2^)**	471	266	444
**^b^SA_pol_ (A°^2^)**	93	66	29
**^c^SA_polre_**	0.197	0.248	0.065
**LogP**	3.22	3.49	10.44
**MW (g/mol)**	368	250	430

*The parameters were obtained from ChemBank (http://chembank.broadinstitute.org/).

a: total surface area (SA_tot_), b: polar surface area (SA_pol_), c: relative polar surface area (SA_pol_/SA_tot_).

### Galvinoxyl radical scavenging

**Figure 2:**
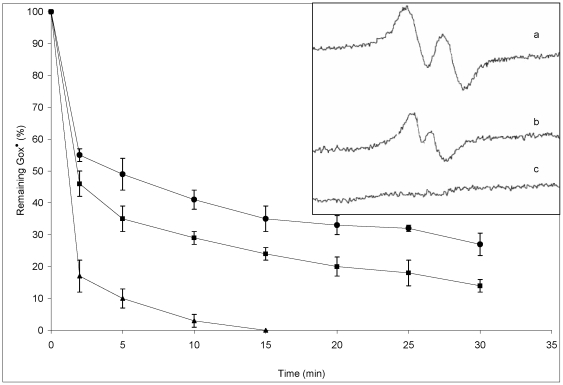
Evaluation of Gox^•^ scavenging rate by curcumin based on ESR results. The ESR spectra were followed after addition of different concentrations of curcumin (10 µM •; 20 µM ▪; 40 µM ▴). Inset: ESR spectra of 10 µM galvinoxyl radicals in different conditions. a) before addition of curcumin, b) 10 min after addition of 10 µM curcumin, c) 10 min after addition of 40 µM curcumin.

**Figure 3:**
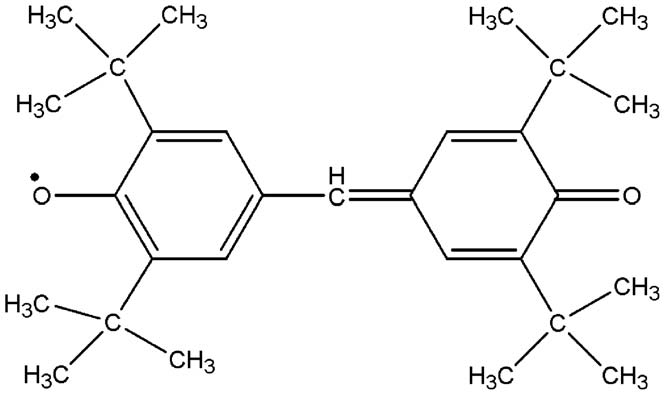
Chemical structures of galvinoxyl radical.

To further confirm the aforementioned findings, we studied the free radical scavenging ability using Galvinoxyl radical (Gox^•^). It is known as a standard free radical for antioxidant studies [28]. Free radical molecules of Gox^•^ are easy to detect by ESR techniques because they have unpaired single electrons. The ESR spectra of Gox^•^ in ethanol are shown in the inset of Figure 2. The height of the ESR signal is directly proportional to the concentration of the Gox^•^ radicals. When the levels of this radical are reduced by curcumin, the spectrum signal disappears (see the spectra b and c). Chemical reduction of Gox^•^ free radicals by curcumin generates GoxH that has a paired electron. Figure 2 shows that the reduction of Gox^•^ free radicals by curcumin is the time and concentration dependent phenomenon. Within 10–15 min, Gox^•^ radicals are completely reduced to GoxH in the presence of 40 µM of curcumin. But intracellular ROS assessment showed that curcumin was highly efficient against intracellular radicals. Comparing the results of Figure 2 with Figure 1, curcumin is less active to reduce Gox^•^. This is probably because of the steric hindrance among adjacent groups within a Gox^•^ molecule. It was already shown that the methoxy substitution in flavonoids introduced steric hindrance, resulting in significant reduction in the rate constant with DPPH radicals [29]. Gox^•^ molecule has high steric resistance because of the size of groups within a molecule as shown in Figure 3. Hence, steric hindrance within a Gox^•^ molecule prevents chemical reactions with curcumin that are effectively observed in smaller oxidative molecules of H_2_O_2_, HO^•^, ROO^•^. Here, steric factor is responsible for such drastic changes in the Gox^•^ radical scavenging ability of curcumin.

### Electron-transfer ability and comparative physical properties of curcumin

**Figure 4:**
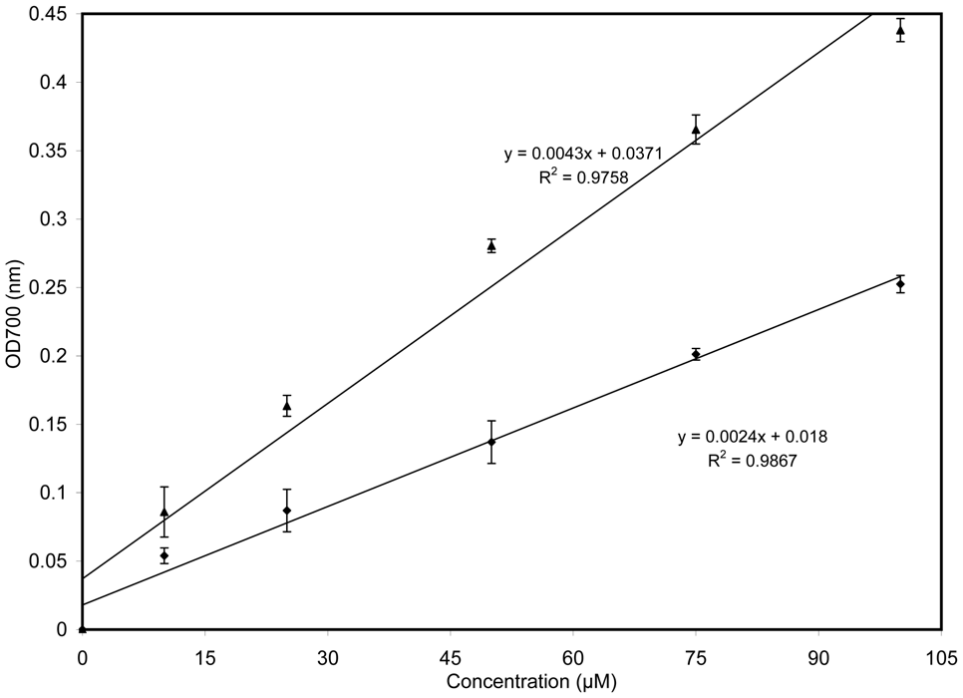
Ability of curcumin to reduce ferric iron in comparison with a known hydrophilic antioxidant, trolox. Measurements were made at pH 7.4 in phosphate buffered saline (PBS) using a UV-Vis spectrophotometer to monitor the reduction of ferric ion to ferrous ion at OD_700_ nm. This assay used a 1∶1 mixture of potassium ferricyanide (1%) and PBS containing different concentrations of curcumin (▴) and trolox (♦).

**Figure 5:**
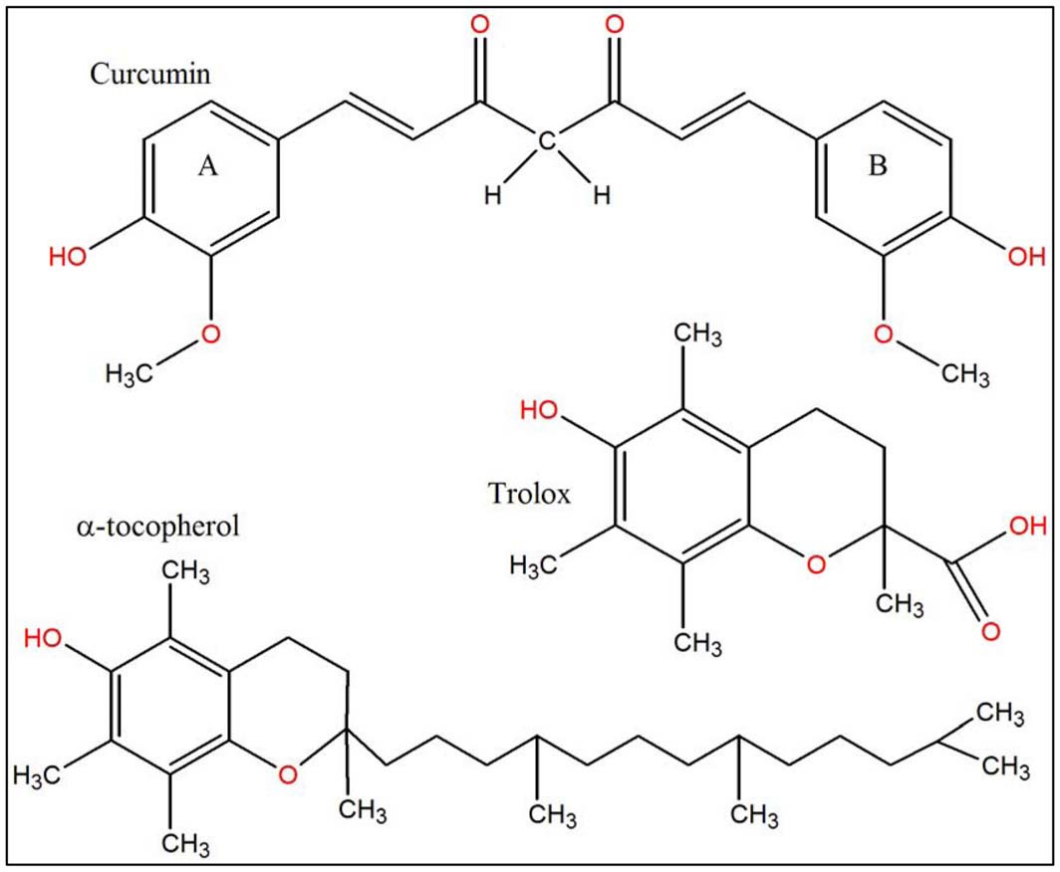
Chemical structures of curcumin, α-tocopherol, and trolox.

The ability of a compound to carry out the reduction of Fe(III) to Fe(II) is a significant indicator of its antioxidant activity [30]. Figure 4 shows a good linear relationship between curcumin concentrations and reduced amounts of Fe^3+^ ions in polar PBS solution. It also indicates that curcumin has almost a two-fold higher reduction potency compared with trolox under the same conditions. Figure 5 may explain the reason of higher reducing potency of curcumin. Curcumin has a unique conjugated structure including two methoxylated phenols and a β-diketone. Generally, the antioxidant compounds can be categorized into two series: phenolics and β-diketones [31]. Compounds in the first series act primarily as hydrogen atom donators while those in second series act as electron donors, leading to chain-breaking reactions of radicals. Consequently, curcumin shows a higher reducing capability because of its unique structure and functional groups, including a β-diketo group and phenyl rings that contain more π electrons than trolox (see Figure 5). The π electrons can fully conjugate between benzene units; this endows them with a high reducing capability. As a result, curcumin is a good electron transfer antioxidant that is active in polar solvents.

The water insolubility of α-tocopherol limited the possibility of comparing and assessing the reducing potency of this compound and curcumin. Trolox (an analogue of Vitamin E) is usually used instead of α-tocopherol (Vitamin E) in antioxidant studies because of its solubility in polar solvents and its high stability. Curcumin is inherently a lipophilic compound, but compared to α-tocopherol, it dissolves in water at low concentrations. Table 2 shows some of the main properties of curcumin, trolox, and α-tocopherol. Because curcumin has more oxygen groups (H-bond donor/acceptor potency) in its structure, its solubility in aqueous media is three times greater than that of α-tocopherol (compare LogP values). Calculating the “relative polar surface area” (SA_polre_) from Table 2 indicates that curcumin has a three times more SA_polre_ than α-tocopherol. These special physical-structural properties endow curcumin with the capability of scavenging intracellular ROS and serving as an electron transfer in aqueous polar media, whereas α-tocopherol is unable to do so. Moreover, in α-tocopherol and trolox, the β-diketone group is absent, therefore the high electron transfer in curcumin should correspond to the β-diketone group.

### Comparative antioxidant mechanism of curcumin

**Figure 6:**
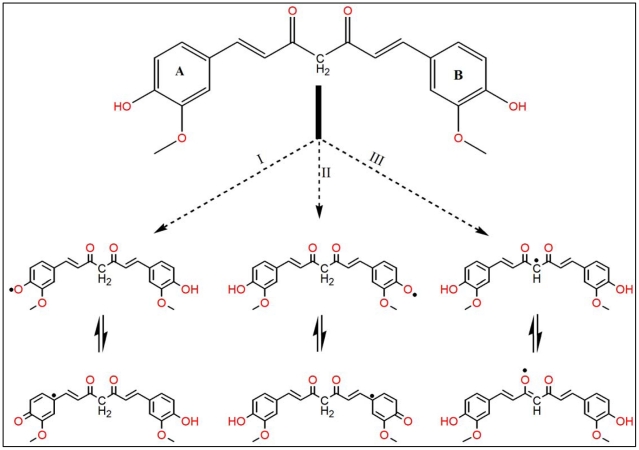
The mechanism of probable three different sites of curcumin reactions with free radicals. Reactions I and II produce a phenoxyl radicals and reaction III produces the carbon-centered radical at the methylene CH_2_ group.

The antioxidant activity of curcumin to inhibit lipid peroxidation has been reported as being more powerful than that of α-tocopherol [32]. Our present results also indicated that curcumin was two fold more effective at iron reduction when compared to trolox. Recently, we showed that the antioxidant potential of the phenolic compounds such as trolox depends on the number and position of phenolic OH groups [30]. As indicated in Figure 5, curcumin has two o-methoxy phenolic OH groups attached to the β-diketone moiety having methylene CH_2_ group. It is believed that the H abstraction from these groups are responsible for the remarkable antioxidant activity of curcumin. Some methods attributed the H abstraction from the methylene CH_2_ group [33], [34] but others attributed to the phenolic OH groups [35], [36]. In Figure 6 it was illustrated the mechanism of probable two sites of free radical reaction with curcumin to produce a phenoxyl radicals (reaction I and II) or at the methylene CH_2_ group to produce the carbon-centered radical (reaction III). These radicals are resonance stabilized and can be interconverted through the conjugation. Therefore, curcumin has two phenolic O-H groups and one methylene CH_2_ group that are capable of H bond-dissociation enthalpy, while trolox has only one phenolic O-H. Therefore, the presence of two identical OH groups as well as methylene CH_2_ group in curcumin, which compared to trolox, can easily undergo successive oxidations. The free radical scavenging activity of curcumin can arise by the resonance stabilization of its radicals from two phenolic OH groups (mainly) or from the CH_2_ group of the β-diketone moiety (Figure 6). Therefore, curcumin not only is a phenolic antioxidant that mostly donates H atoms from the phenolic groups, but also is a β-diketone radical chain-breaking substance that can give H atom from methylene CH_2_.

All together, curcumin can undergo either “electron transfer” and/or “H-atom donation” to react with free radicals. Three different functional dissociable groups and a β-diketone site are important factors responsible for higher antioxidant potency of curcumin with excellent reducing power rather than that of trolox.
